# Malignant primary female genital system lymphoid

**DOI:** 10.3389/fonc.2024.1438812

**Published:** 2024-09-26

**Authors:** Qiucheng Jia, Huimin Tang, Zhiyong Dong, Wanying Chen, Mengyue Chen, Weiwei Wei, Jiming Chen

**Affiliations:** ^1^ Department of Gynecology, The Affiliated Changzhou Second People’s Hospital of Nanjing Medical University, Changzhou, China; ^2^ Suqian First People’s Hospital of Nanjing Medical University, Suqian, Jiangsu, China; ^3^ Department of Obstetrics and Gynaecology, The Second Affiliated Hospital of Chongqing Medical University, Chongqing, China

**Keywords:** non-Hodgkin’s lymphoma, primary female genital system lymphoma, uterine lymphoma, ovarian lymphoma, vulvar lymphoma

## Abstract

Lymphoma is a malignant tumour of the lymphatic system with an incidence rate of about 6.6 per 100,000 people. Among the many lymphoma types, the most common is non-Hodgkin’s lymphoma. Lymphomas are common in the gastrointestinal tract, breast, neck, etc., while those in female genital tracts are rare. In this article, we report four cases of primary female genital system lymphoid malignancies diagnosed and treated at our hospital from 2018 to 2023, with a systematic review.

## 1 Background

Lymphoma is a general term for a group of malignant tumours of the lymphohematopoietic system with an incidence rate of approximately 6.6 per 100,000 people. Specifically, 90% of them are non-Hodgkin’s lymphomas (1). Non-Hodgkin’s lymphoma can occur in or outside the lymph nodes and usually presents as a progressively enlarged, painless mass. The most lymphoma-prone sites are the gastrointestinal tract, neck, and central nervous system, but rarely the lymphoma may also occur in sites such as liver, lung, and bladder. Approximately 0.5% to 1.5% of extranodal lymphomas are found in the female reproductive system, of which 75% are of the primary type (1, 2). According to statistical analysis, female genital lymphomas are most commonly found in the ovary, cervix, and uterus (2). We will report one case of primary uterine lymphoma with uterine lesions as the first symptom, two cases of asymptomatic primary ovarian lymphoma, and one case of vulvar lymphoma (See **Tables 1**, **2** for details).

**Table 1:** Clinical characteristics, laboratory investigations, surgical approach and postoperative pathology in four patients.

Case	Age	Clinical characteristic	Lesion location	Lesion diameter	LDH (U/L)	CA125	Surgery/tissue biopsy	Pathological type
1	66	postmenopausal vaginal bleeding for 1 year	uterine	Uterus (9.0*7.8*8.6cm), uterine cavity (4.9*0.7cm)	264	14.26	hysteroscopy	Diffuse large B lymphoma
2	40	adnexal mass gradually increasing for 1 year	Right ovary	Right ovary (7.1*4.7 cm)	195	22.23	Laparoscopic unilateral ovariectomy	Diffuse large B lymphoma
3	58	discovery of gradual enlargement of bilateral adnexa for 6 months	bilateral ovaries	Left ovary (4.5*3.5*3.5cm), right ovary (7.5*7*4cm)	257	21.58	Laparoscopic Bilateral Oophorectomy	Diffuse large B lymphoma
4	77	1-month-old vulvovaginal mass with swelling and pain	vulva	5.7cm*2.9cm	97.7	17.73	excision of vulvar lesions	Diffuse large B lymphoma

**Table 2:** Postoperative immunohistochemistry, PET-CT, staging, treatment and prognosis in 4 patients.

Case	Immunohistochemistry	PET-CT	Staging (Ann Arbor staging system)	Chemotherapy	Follow up
1	CD20 (+), CD79a (+), CD3 (-), CD5 (-), KI-67 (+, 80%), BCL-2 (+, 80%), cD10 (+), Bcl-6 (+), Mum-1 (+), c-Myc (+, 30%), EBER (-), EBER (-), EBER (+, 30%) EBER (-), CD21 (-), CyclinD1 (+), CD23 (-)	1.Enlarged uterus with increased glucose metabolism, malignant lesions were considered. Follow-up was recommended in the left external iliac paravascular and both inguinal lymph nodes.2.Systemic bone marrow diffuse increased glucose metabolism	II	R-CHOP*6	12 months after surgery, survived, no signs of recurrence
2	CD20 (+) CD79α (+) Pax5 (+) CD3 (partly +) CD5 (partly +) Ki-67 (+, 40%) bcl-2 (+) bcl-6 (+) CD10 (+) Mum-1 (-) CD23 (+) C-myc (+, 5%) EBER (-) CKp (-) EMA (-) CD30 (+) CD15 (-) CyclinD1 (-) CD4 (partial +) CD8 (partial +) CD43 (partial +) ALK-Ventana D5F3 (-)	1. postoperative changes of uterine fibroids and right ovarian tumour debulking; pelvic effusion; 2. multiple retroperitoneal lymph node shadows with fuzzy thickening of the fat interstitial space in the root of the mesentery, measuring approximately 1.6x0.9 cm, with a maximum SUV value of approximately 1.8	IV	R-CHOP*6	25 months after surgery, survived, no signs of recurrence
3	CD20 (+), Bcl-2 (+, 70%), C-myc (+, 10%), Bcl-6 (+), CD3 (-) Ki-67+ (+, 80%), MUM (+), CD10 (-)	bilateral postoperative ovarian lymphomas with retroperitoneal and bilateral iliac paravascular lymph node invasion	IV	R-CHOP*6	67 months after surgery, survived, no signs of recurrence
4	CK (-), CD20 (++++),CD5 weak (+), CD79a (++++), Ki67 (+, 90%), BCL-2 (+, 90%), Bcl-6 (+, 60%), CD10 (+, 50%), Mum-1 (+, 60%), c -Myc (+, 40%), CD23 (-), P53 (+, mutant), EBER (-)	After resection of the right vulvar lesion, bilateral inguinal and pelvic bilateral iliac paravascular enlarged lymph nodes with abnormally increased glucose metabolism were considered a high probability of lymphoma involvement	IV	Recommended chemotherapy treatment, no chemotherapy for now	5 months after surgery, currently in stable condition

## 2 Case presentation

### 2.1 Case 1

**Figure 1:**
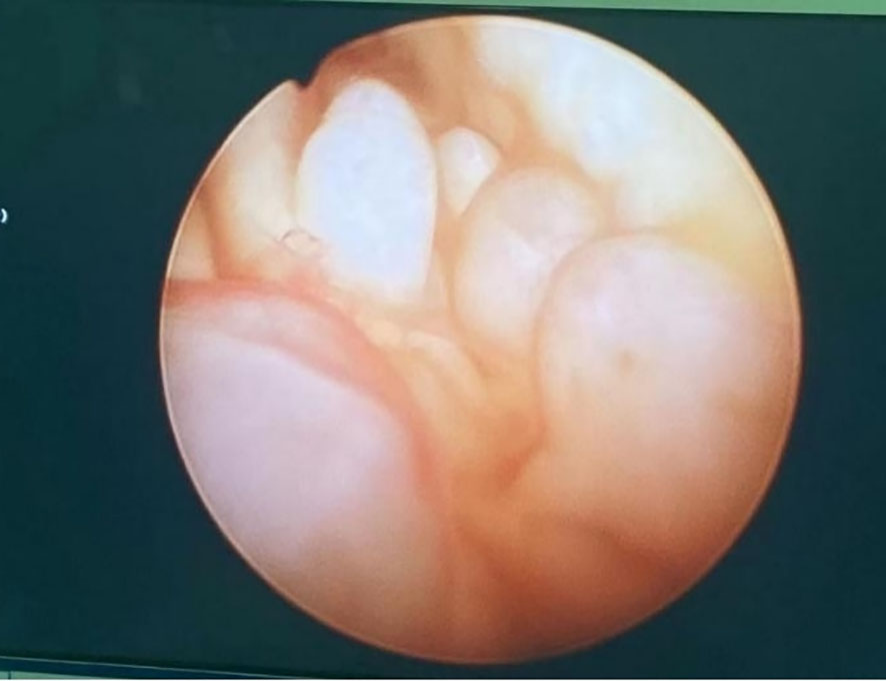
Hysteroscopic view of the uterine cavity.

The patient, female, 66 years old, was admitted to the hospital because of “postmenopausal vaginal bleeding for 1 year”. The ultrasound showed that the uterus was about 9.0×7.8×8.6 cm in size, and the mixed echoes in the uterine cavity were about 4.9×0.7cm. The relevant examinations before hospitalisaiton were completed, and the lactic acid dehydrogenase was 264 U/L. Other examinations such as tumour indexes did not show any obvious abnormality. The patient underwent hysteroscopy at our hospital, during which the uterine cavity was found to be filled with dark red cauliflower-like tissue (see **Figure 1**). After diagnostic scraping, the sample was sent to pathology.

**Figure 2:**
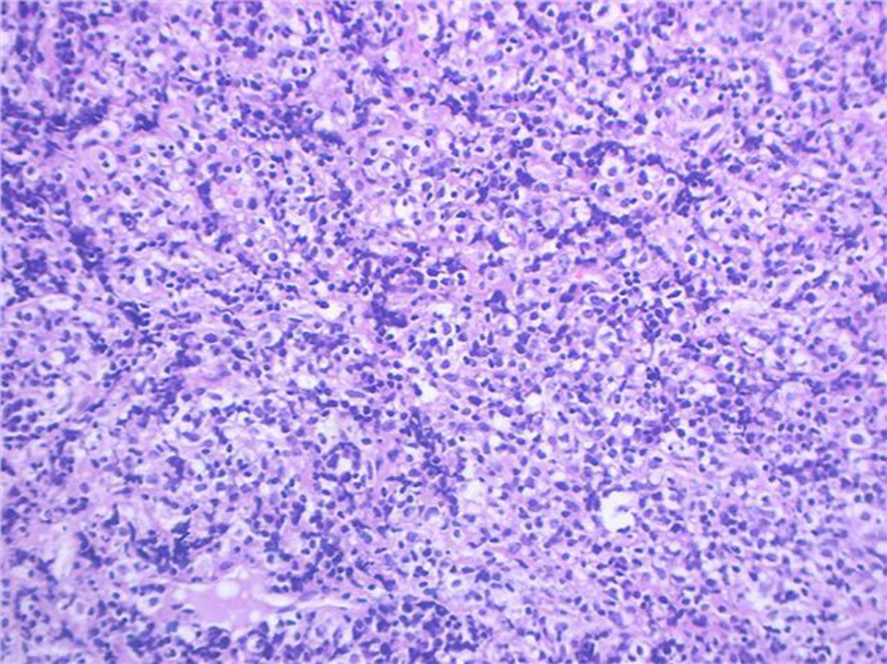
Intrauterine diagnosis and pathology.

Pathology + immunohistochemistry: Diffuse tumour cells with a predilection for the lymphohematopoietic system (see **Figure 2**). cD20 (+), cD79a (+), cD3 (-), cD5 (-), KI-67 (+, 80%), BCL-2 (+, 80%), cD10 (+), Bcl-6 (+), Mum-1 (+), c-Myc (+, 30%), EBER (-), EBER (-), EBER (+, 30%) EBER (-), CD21 (-), CyclinD1 (+), CD23 (-).

**Figure 3:**
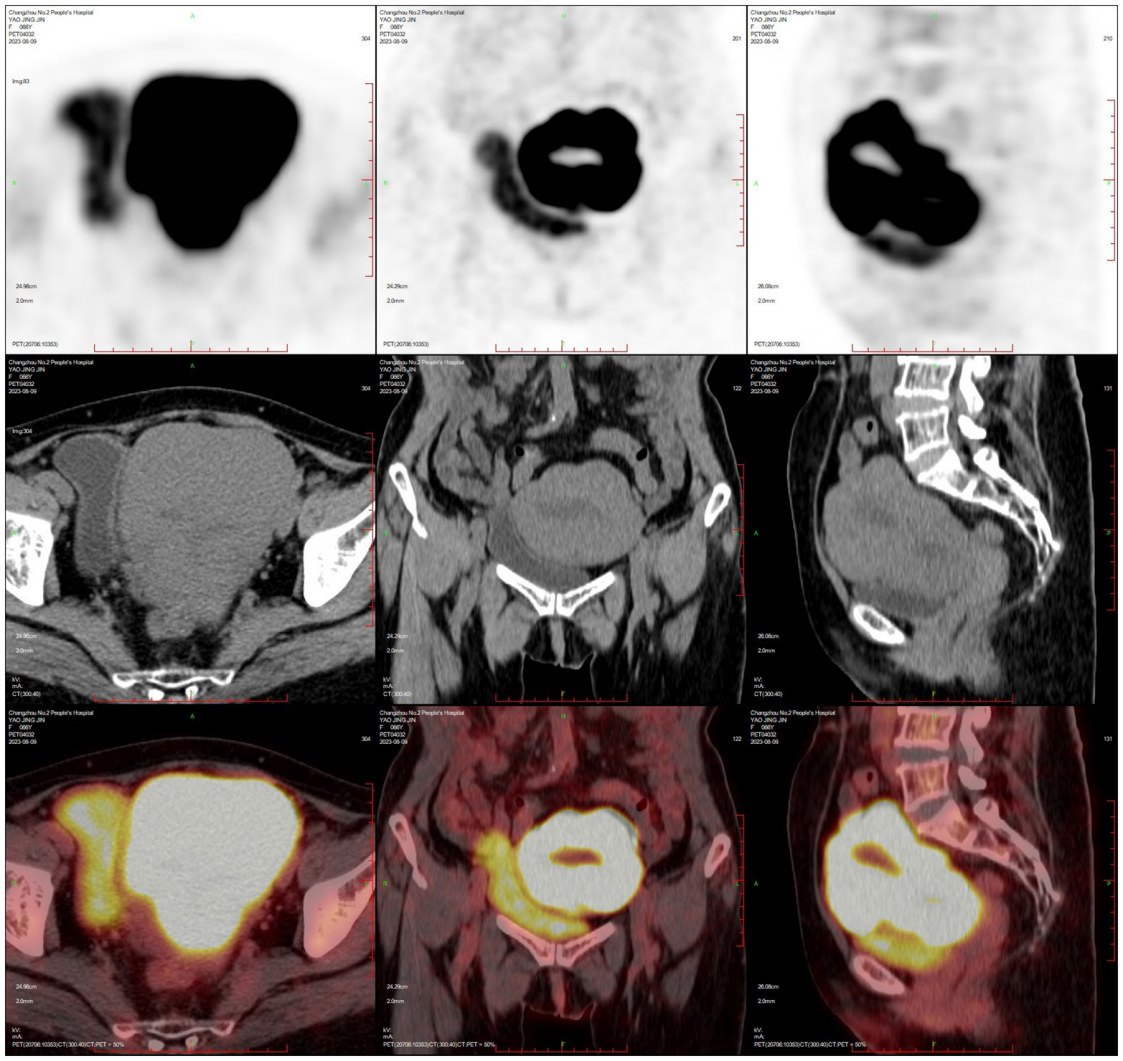
Uterine lymphoma PET-CT.

PET-CT (after hysteroscopy): 1. Enlarged uterus with increased glucose metabolism, and malignant lesions were considered. Follow-up was recommended in the left external iliac paravascular and both inguinal lymph nodes. 2. Systemic bone marrow diffuse increased glucose metabolism (see **Figure 3**).

The patient underwent immunochemotherapy with the R-CHOP regimen in our hematology department. The specific regimen was: rituximab 600mgd0, cyclophosphamide 0.8g d1, epirubicin 80mg d1, vinblastine 3mg d1, and prednisone 40mg bid d1-d5. She has now had six courses of chemotherapy and is stable. There was no evidence of relapse at the one-year reexamination after chemotherapy.

### 2.2 Case 2

The patient, female, 40 years old, was admitted to the hospital on 2022-01-16 because of “adnexal mass gradually increasing for 1 year”. The ultrasound showed that there were multiple fibroids in the uterus and an uneven mass of 7.1×4.7cm in the right adnexal region. The relevant examinations before hospitalisaiton were completed. LDH: 195 U/L. No obvious abnormality in other investigations such as tumour index was found. Laparoscopic myomectomy + ovarian tumour debulking was performed.

**Figure 4:**
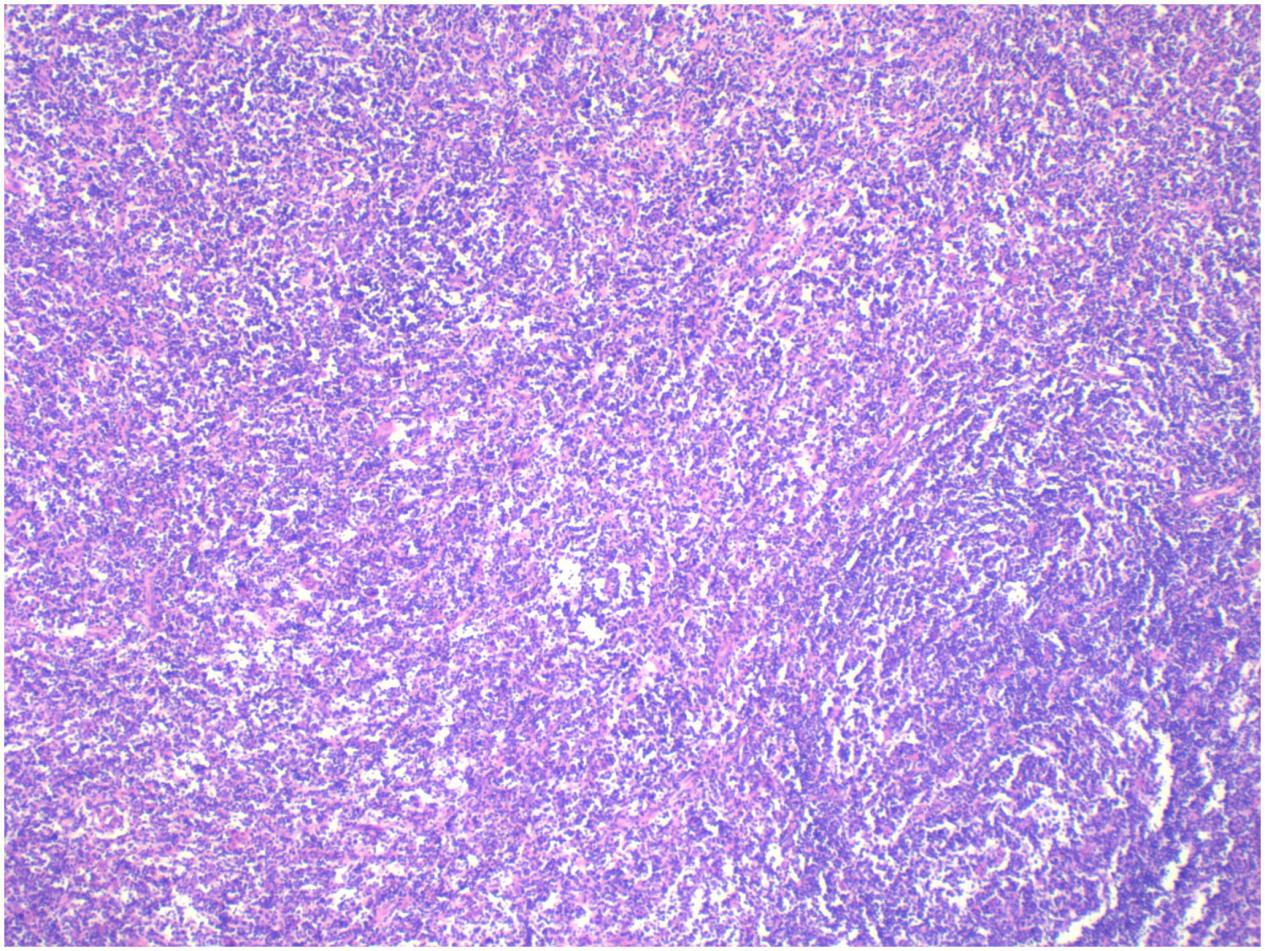
Postoperative pathology of the left ovary.

Pathology: The right ovarian tumour was considered to be malignant lymphoma, diffuse large B-cell type, with a T-cell-rich background (see **Figure 4**).

**Figure 5:**
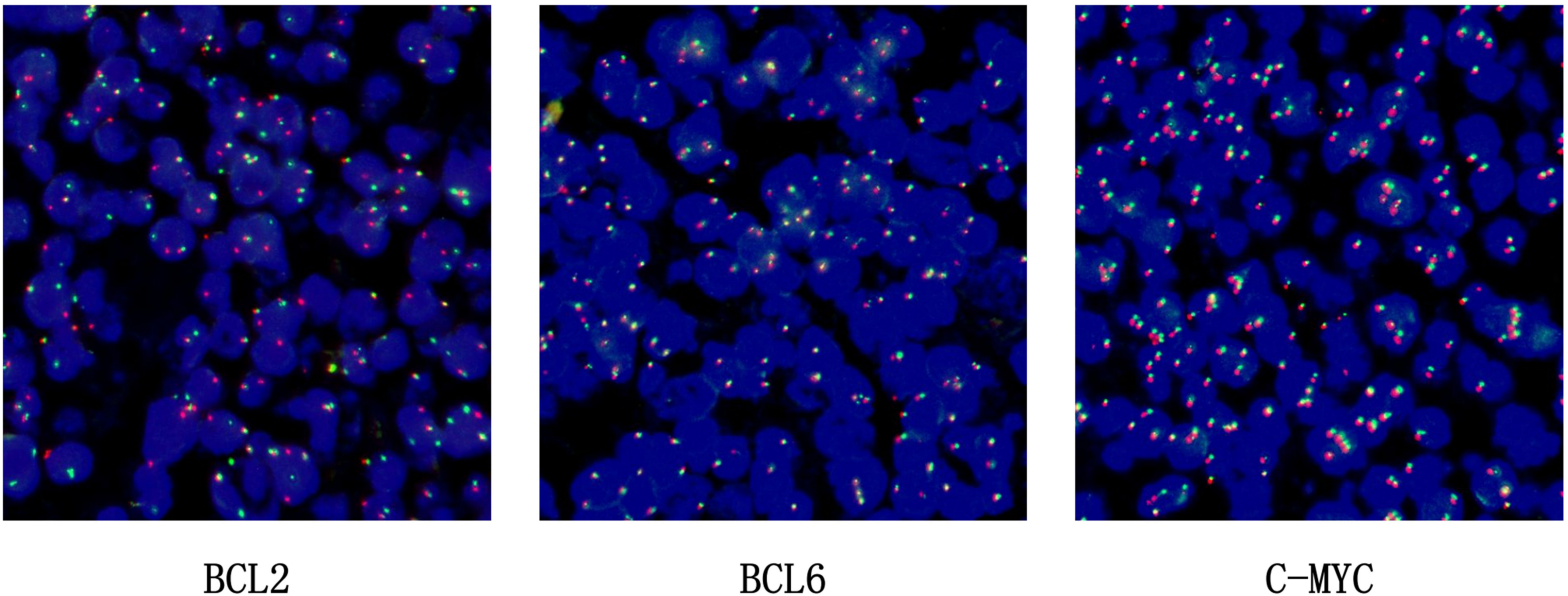
FISH for BCL-2, BCL6, C-MYC.

BCL2 probe: The BCL2 gene in the tumour cells showed a ratio of one red, one green, and one yellow accounting for more than 30%, indicating that the BCL2 gene had been broken and recombined. BCL6 showed no obvious abnormality and the c-MYC gene was amplified, not broken and recombined (see **Figure 5**).

Immunohistochemistry: CD20 (+) CD79α (+) Pax5 (+) CD3 (partially +) CD5 (partially +) Ki-67 (+, 40%) bcl-2 (+) bcl-6 (+) CD10 (+) Mum-1 (-) CD23 (+) C-myc (+, 5%) EBER (-) CKp (-) EMA (-) CD30 (+) CD15 (-) CyclinD1 (-) CD4 (partially +) CD8 (partially +) CD43 (partially +) ALK-Ventana D5F3 (-).

Ovarian pathology section Genotyping - IGH + genotyping TCRD + fusion gene - rearrangement: IGH gene rearrangement: positive; IGK gene rearrangement: positive; TCRβ gene rearrangement: positive.

**Figure 6:**
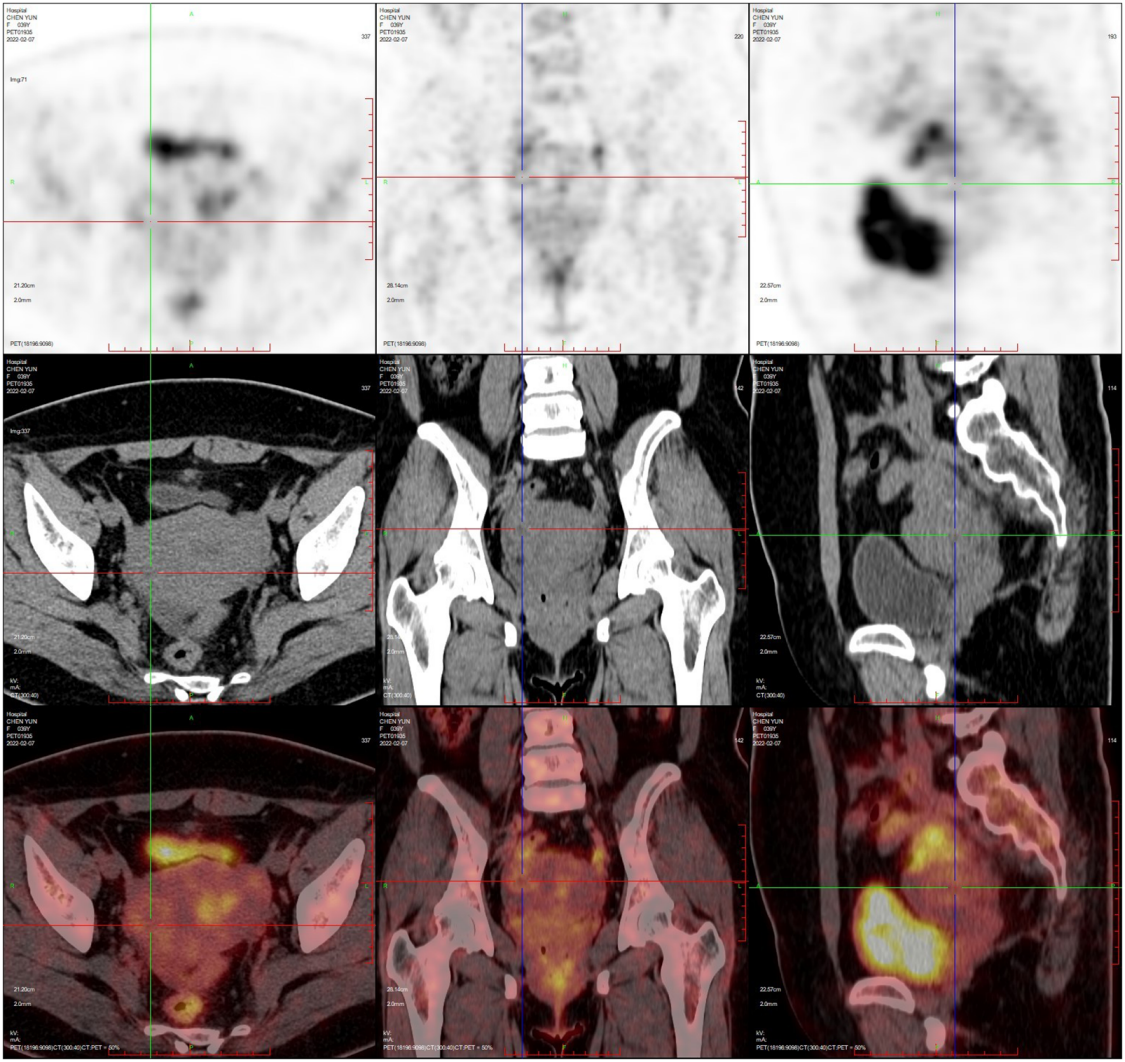
PET-CT after ovarian lymphoma surgery (Case 2).

PET-CT (postoperative): 1. Postoperative changes of uterine fibroids and right ovarian tumour debulking; pelvic effusion; 2. Multiple retroperitoneal lymph node shadows with fuzzy thickening of the fat interstitial space in the root of the mesentery, measuring approximately 1.6×0.9cm, with a maximum SUV value of approximately 1.8 (see **Figure 6**).

The patient underwent six cycles of R-CHOP intravenous immunochemotherapy in the hematology department within 6 months of surgery. The specific regimen was rituximab 700mg d0, epirubicin liposome 120mg d1, vincristine 4mg d1, cyclophosphamide 1.35g d1, and prednisone 50mg bid d1-5. The patient’s lymphoma was stable after two years of regular check-ups at our hospital, with no signs of recurrence.

### 2.3 Case 3

**Figure 7:**
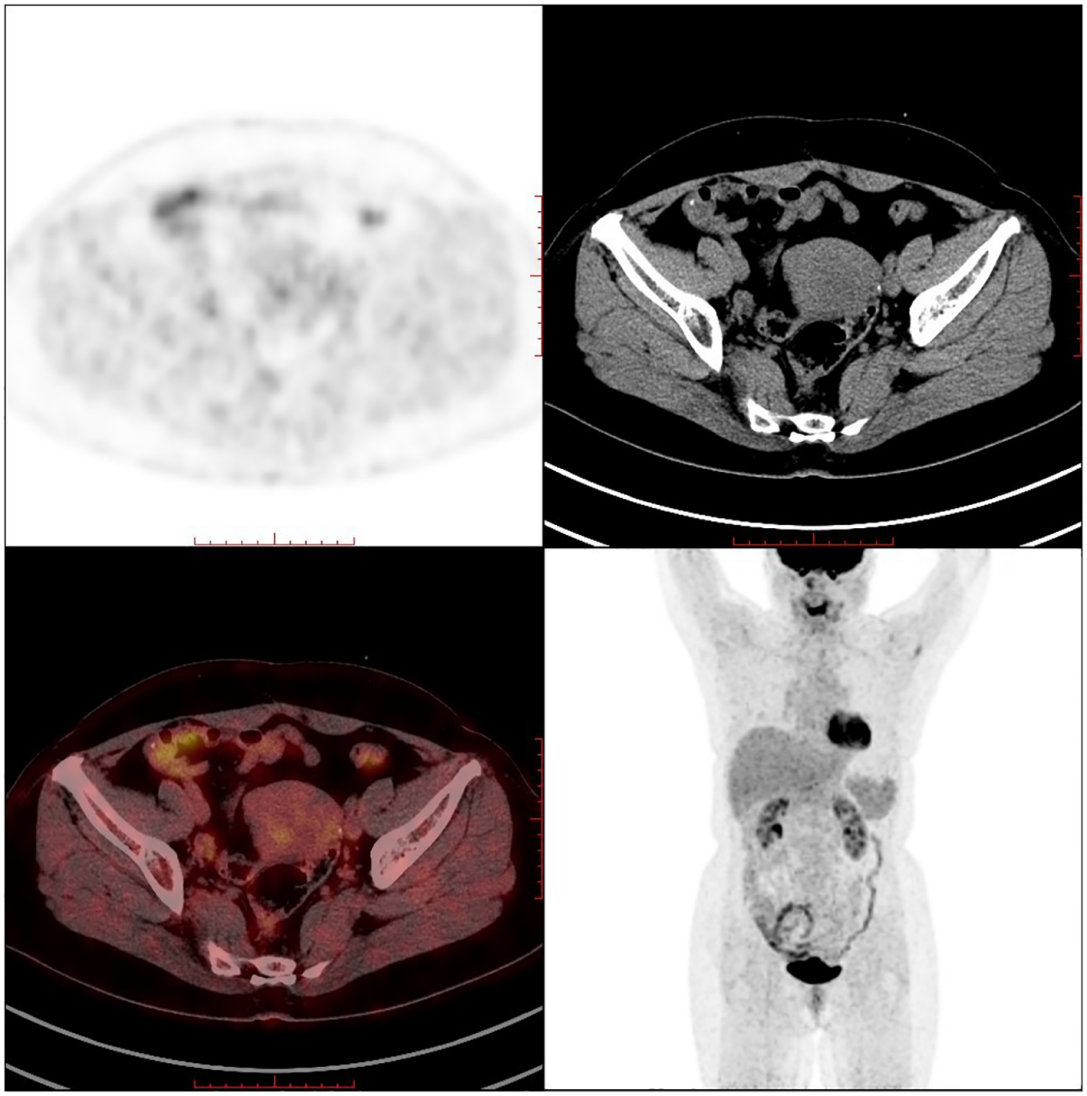
PET-CT after ovarian lymphoma surgery (Case 3).

The patient, female, 58 years old, underwent ovarian tumour subtraction in our hospital on 2018-06-13 due to “discovery of gradual enlargement of bilateral adnexa for 6 months”. Postoperative pathology: Left ovary 4.5×3.5×3.5cm, right adnexa 7.5×7×4cm, which is in line with diffuse large B lymphoma symptom. Immunohistochemistry: Tumour cells CD20 (+), Bcl-2 (+, 70%), C-myc (+, 10%), Bcl-6 (+), CD3 (-) Ki-67+ (+, 80%), MUM (+), CD10 (-). FISH: No c-MYC gene translocation, no Bcl-2 gene translocation, and BCL6 gene related translocation. The patient received postoperative PET-CT re-examination in our hospital: After the bilateral ovarian lymphoma surgery, retroperitoneal and bilateral iliac paravascular lymph nodes were invaded (see **Figure 7**).

The patient underwent six times of R-CHOP intravenous immunochemotherapy in the hematology department within 6 months of surgery. The specific regimen was rituximab 600mg d0, doxorubicin hydrochloride liposome 81.5mg d1, vincristine 3mg d1, cyclophosphamide 1.22g d1, and prednisone 50mg bid d1-5. The patient has been followed up regularly in our hospital for three years after chemotherapy, and no recurrence of lymphoma has been observed, and her current lymphoma status is stable.

### 2.4 Case 4

**Figure 8:**
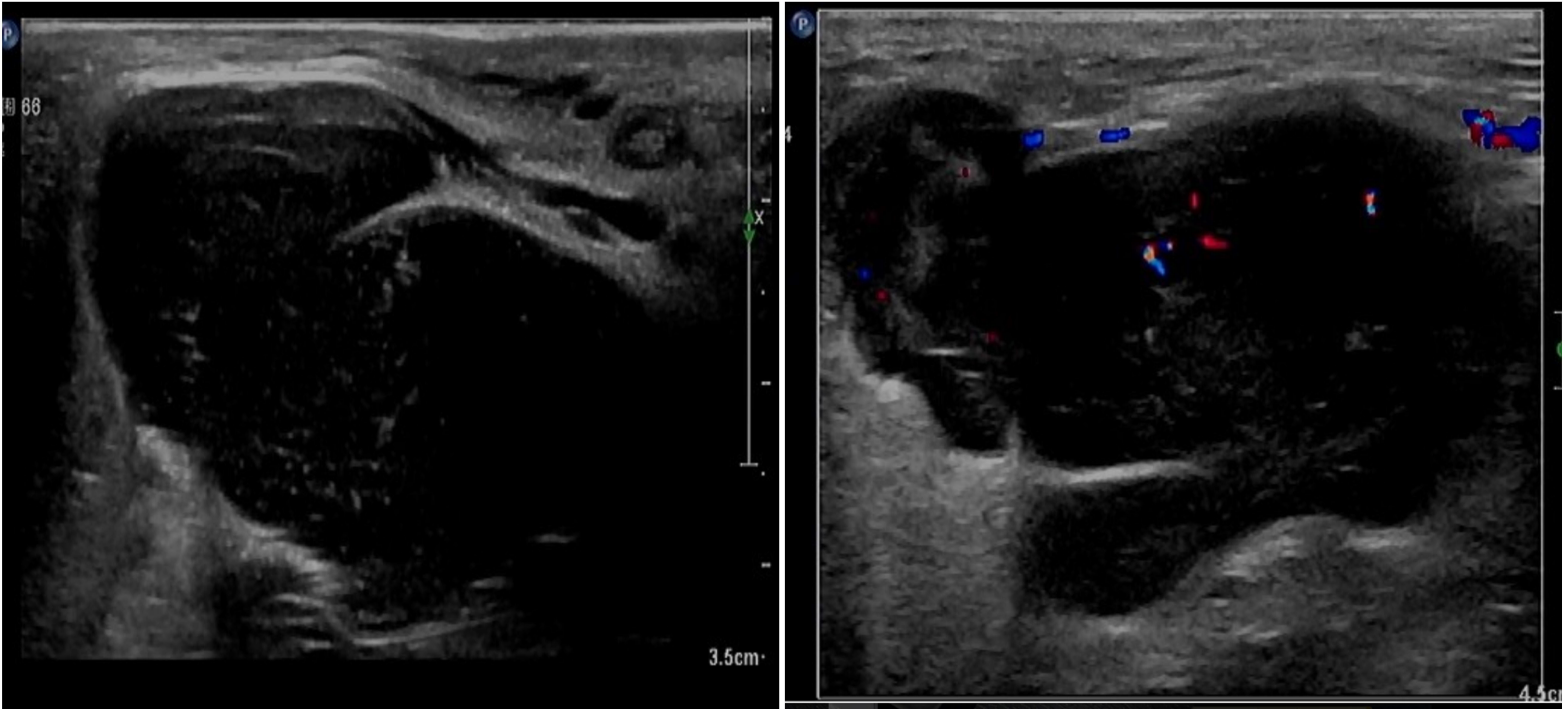
Perineal superficial mass through CDU.

**Figure 9:**
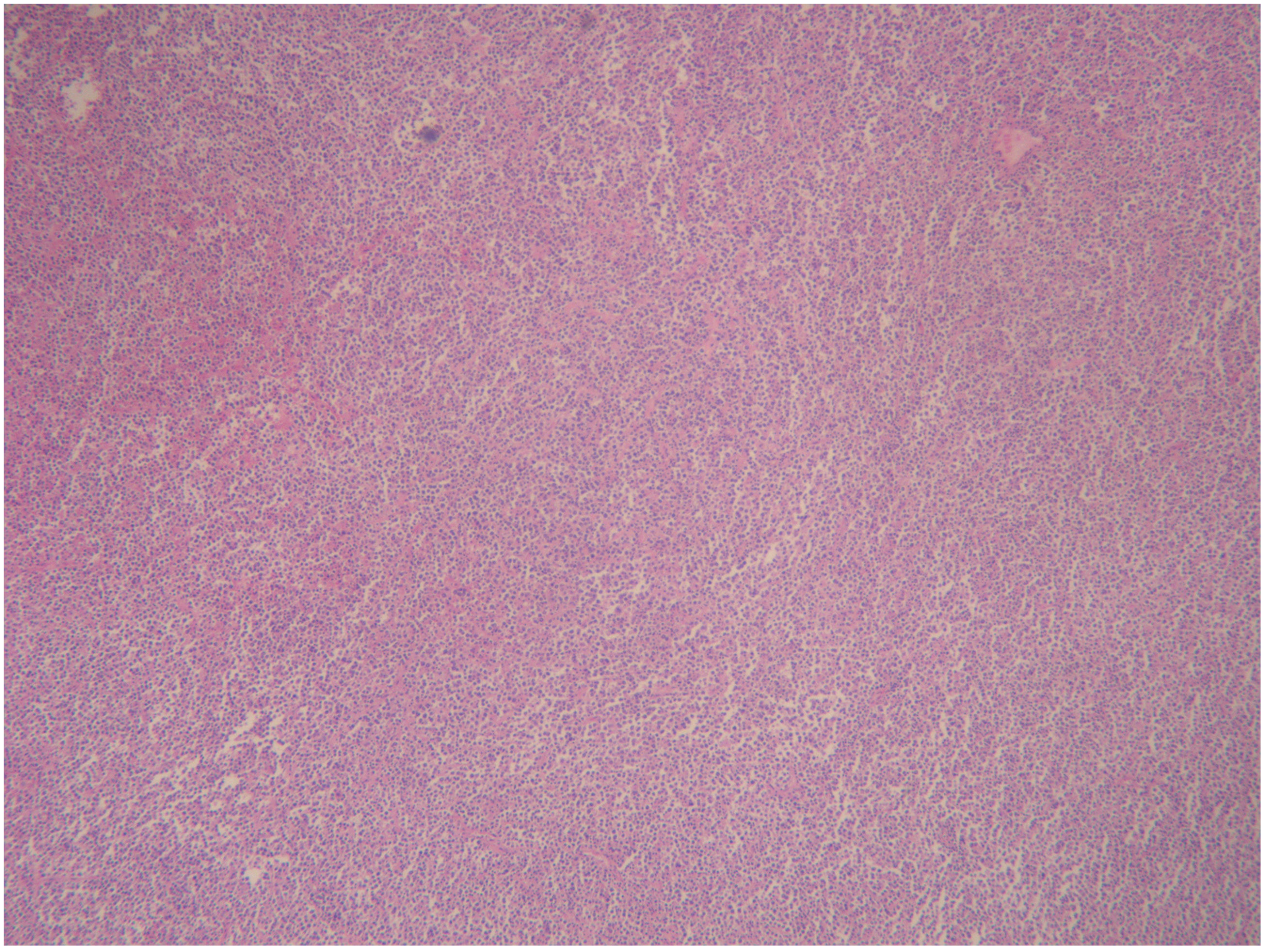
Diffuse large B cell lymphoma (DLBCL) on vulva.

**Figure 10:**
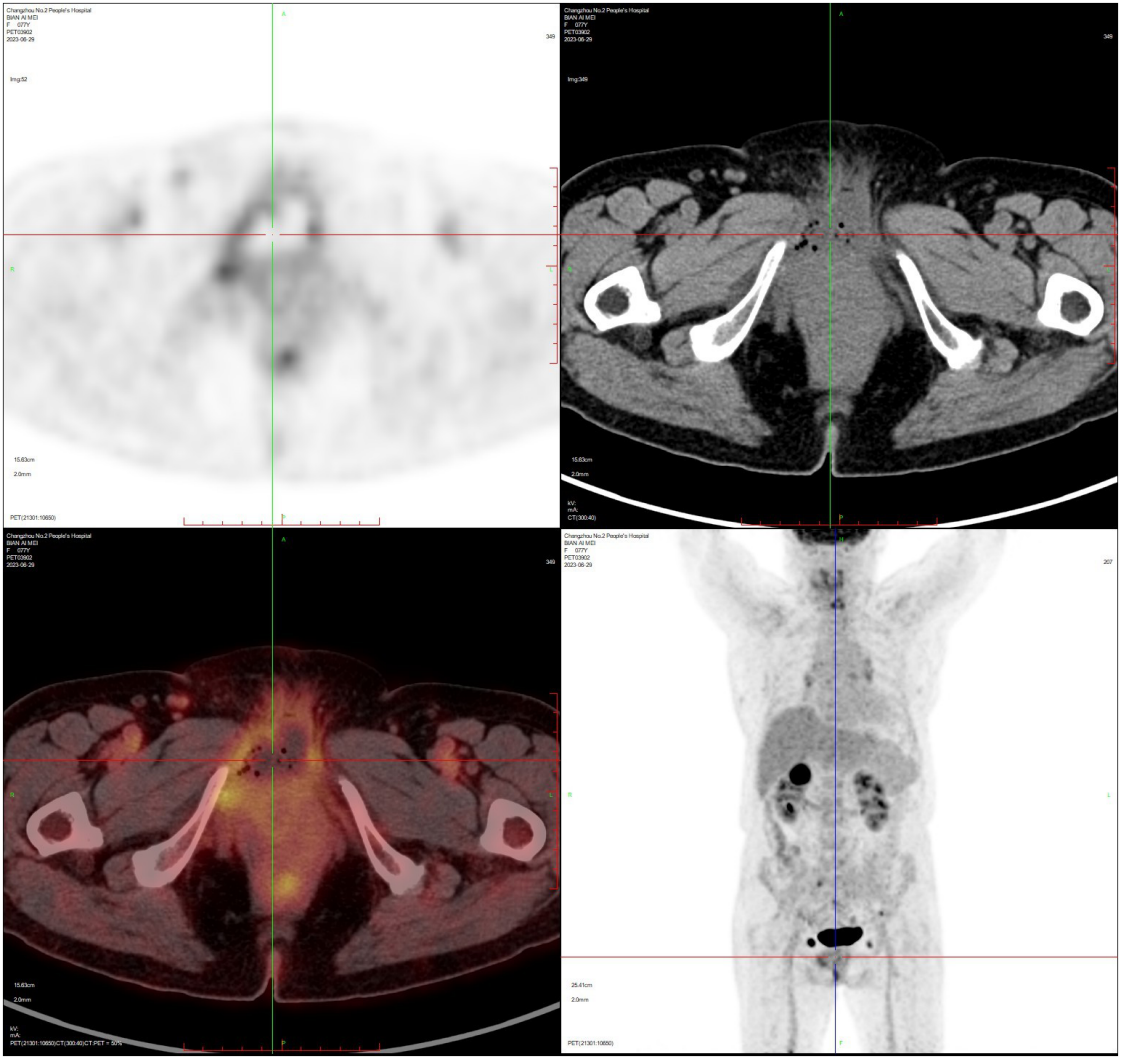
PET-CT after vulvar lymphoma surgery.

The patient, female, 77 years old, underwent vulvovaginal lesion excision in our hospital on 2023-06-13 for a “1-month-old vulvovaginal mass with swelling and pain”. Preoperative superficial mass (see **Figure 8**): Mixed echoes of about 5.7×2.9cm in size were seen under the perineal skin and blood flow signal was visible. Intraoperatively, a mass of about 4×4×4.5cm was seen on the external labia majora of the mons pubis, with a hard texture, poor mobility, and deep to the surface of the descending branch of the pubic bone and the pubic symphysis. After complete excision of the mass, a section view showed that the section surface was fish-like with a brittle texture. Postoperative pathology: (Vulvar mass) Highly aggressive B-cell lymphoma (see **Figure 9**), immunohistochemistry: CK (-), CD20 (++++), CD5 weak (+), CD79a (++++), Ki67 (+, 90%), BCL-2 (+, 90%), Bcl-6 (+, 60%), CD10 (+, 50%), Mum-1 (+, 60%), c-MYC (+, 40%), CD23 (-), P53 (+, mutant), EBER (-). Postoperative PET-CT (see **Figure 10**): After resection of the right vulvar lesion, the patient’s bilateral inguinal and pelvic bilateral iliac paravascular enlarged lymph nodes showed abnormally increased glucose metabolism, which was considered lymphoma involvement of a high probability.

## 3 Discussion

Lymphoma is a malignant tumour that originates in the lymph nodes or extra-nodal lymphoid tissues, and can be classified according to its pathology into Hodgkin’s lymphoma and non-Hodgkin’s lymphoma. It can occur in a variety of sites and in any organ of the body. Studies have shown that about 30% of non-Hodgkin’s lymphomas occur outside the lymph nodes and are most commonly seen in the gastrointestinal tract and neck (3). Non-Hodgkin’s lymphomas originating in the female reproductive system accounts for only 0.5%–1.5% of extranodal cases, including the ovary (37%), cervix (21.4%), uterus (16.5%), vagina (11.8%), and vulva (8.8%) (4). Currently, the criteria proposed by Vang et al. for primary genital lymphoma are still in use: (1) Patient has no previous history of lymphoma. (2) The patient’s primary disease is in the genital system and may involve adjacent genital organs or lymph nodes. (3) Hemorrhagic abnormal cells are present in the peripheral blood or spinal cord. (4) If recurrent lymphoma occurs at a distant site, the time gap between it and the primary lymphoma must be longer than 6 months (5).

### 3.1 Primary ovarian lymphoma

The ovary is the most common site of female genital lymphomas and the clinical presentation is variable. A painless adnexal or pelvic mass that gradually increases in size is usually the first symptom, and the mass is usually large and easily distorted, causing abdominal pain. It may be accompanied by abdominal distension, uterine bleeding, or local symptoms such as frequent urination and constipation due to pressure on surrounding organs. Systemic symptoms such as fever, night sweats, and weight loss may also be present (6). Patients tend to have elevated lactate dehydrogenase and CA125 in most reported cases (7). Histopathology is the gold standard for confirming the diagnosis of ovarian lymphoma. In immunohistochemistry of diffuse large B cell lymphoma (DLBCL) of the ovary, CD10, CD19, CD20, and CD22 are all positive, indicating that B cells are in a high expression status. Besides, the following conditions should be met during the diagnostic period: 1. MYC gene breakpoints and rearrangements. 2. CD10 (+). 3. Ki-67 value at 90%. 4. Absence of BCL-2 expression (8).

Currently, the treatment of ovarian lymphoma is mainly a combination of surgery, chemotherapy, and radiotherapy. Preoperative diagnosis of ovarian lymphoma is usually difficult, so we usually treat it surgically first, and then chemotherapy with R-CHOP regimen after surgery. There remains a big difference between domestic and foreign countries on adoption of complete tumour reduction surgery, and foreign countries believe that surgery plays an important role in providing a diagnostic basis (7). In contrast, the domestic circle believes that reducing the size of the primary lesion as much as possible by removing the uterus, greater omentum, and bilateral fallopian tubes and ovaries will improve the prognosis (9). Primary ovarian lymphoma has a better prognosis. According to a recent study, the 5-year survival rate of patients receiving primary ovarian lymphoma treatment can reach 80% (10). Treatment and prognosis of selected primary ovarian lymphomas are shown in **Table 3** (11–38).

**Table 3:** Some reported cases of primary female genital system lymphoid in recent years.

Case number	Case	Age	Symptom	Location of lesions	Stage	Treatment	Follow up (months)
1	Zainab Jan et al. (11)	18	lower abdominal pain, anorexia, fever	Left ovary	–	underwent hemodialysis two times a week for one month (total of eight cycles).+CHOP*1	1
2	Gerrity C et al. (12)	20	abdominal pain and distension	Left ovary	Ann Arbor stage IVE	Chemotherapy:3 cycles of R-CHOP.	3
3	Madhulika Urella et al. (13)	48	generalized fatigue, lower abdominal discomfort,	Left ovary	Ann Arbor stage IV	total abdominal hysterectomy with bilateral salpingo-oophorectomy+Chemotherapy:6 cycles of DA-EPOCH-R (etoposide, prednisone, vincristine, cyclophosphamide, doxorubicin, rituximab)	18
4	William Sergi et al. (14)	24	abdominal tenderness, diffuse pain, constipation lasting for approximately one month	Right ovary	–	chemotherapy after laparoscopic surgery	–
5	Pei-Chen Li et al. (15)	29	lower abdominal pain	Left ovary	–	laparoscopic staging surgery	–
6	Hongliang Xu et al. (16)	25	Physical examination reveals an ovarian mass	Right ovary	stage I (FIGO)	Right Tubectomy and Oophorectomy+HyperCVAD chemotherapy	18
7	Chanil Deshan Ekanayake et al. (17)	32	Irregular vaginal bleeding	Bilateral ovaries	Ann Arbor stage IVE	Chemotherapy: CHOP*1+UKALL XII(prednisolone, vincristine, daunorubicin, asparaginase, and intrathecally administered methotrexate)*1	Died after two sessions of chemotherapy
8	Giorgio Persano et al. (18)	12	abdominal pain	Bilateral ovaries	stage III (FIGO)	Chemotherapy	–
9	Shilpa Chowdary Peddappolla et al. (19)	11	abdominal pain	Right ovary	stage IA (FIGO)	Lost visits after surgery	–
10	Laila Jaouani et al. (20)	55	pelvic pain	Right ovary	Ann Arbor stage IIE	total hysterectomy with bilateral adnexectomy, omentectomy, appendectomy, and peritoneal biopsies+ R-CHOP*6	36
11	Mehdi Pourghasemian et al. (21)	14	abdominal pain	Bilateral ovaries	Ann Arbor stage II	Chemotherapy: CVAD(cyclophosphamide, vincristine, doxorubicin (Adriamycin), and dexamethasone)+methotrexate plus cytarabine (cytosar) *6	6
12	C. GRIGORIADIS et al. (22)	38	abdominal pain	Bilateral ovaries	Ann Arbor stage IIE	Left salpingo-oophorectomy +CHOP*8	60
13	Rajni Yadav et al. (23)	40	abdominal distension	Bilateral ovaries	Ann Arbor stage II	R-CHOP	7
14		14	pelvic mass	Bilateral ovaries	–	R-CHOP	3
15	Jayant Sastri Goda et al. (24)	52	Post‐menopausal bleeding	cervix	Ann Arbor stage IE	R‐CHOP x 6 + radiotherapy	18
16		50	Post‐menopausal bleeding	cervix	Ann Arbor stage IE	R‐CHOP x 6 + radiotherapy	43
17		39	Foul smelling discharge	cervix	Ann Arbor stage IE	R‐CHOP x 6 + radiotherapy	8
18		62	Post‐menopausal bleeding	cervix	Ann Arbor stage IIE	R‐CEOP x 6 +radiotherapy	10
19	Elena Igwe et al. (25)	22	left lower extremity edema and pelvic pain	cervix	Ann Arbor stage IIE	R‐CHOP	5
20	Mendato et al. (26)	44	Abnormal vaginal bleeding	cervix	Ann Arbor stage IVE	R‐CHOP	24
21	Singh et al. (27)	34	abdominal pain	cervix	Ann Arbor stage IE	R‐CHOP +radiotherapy	60
22	Zhou et al. (28)	31	cervical masses	cervix	Ann Arbor stage IE	R‐CHOP	1
23	Sharma et al. (29)	61	Postmenopausal vaginal bleeding	cervix	Ann Arbor stage IIE	R‐CHOP +radiotherapy	1
24	Regalo et al. (30)	40	Lower limb pain, swelling	cervix	Ann Arbor stage IE	R‐CHOP	45
25	Cubo et al. (31)	51	Postmenopausal vaginal bleeding	cervix	Ann Arbor stage IE	R‐CHOP	24
26	Del M et al. (32)	36	Vaginal bleeding, pelvic pain	cervix	Ann Arbor stage IV	R‐CHOP	15
27	Laila Abarray et al. (33)	66	Postmenopausal vaginal bleeding	cervix	Ann Arbor stage IE	R‐CHOP +radiotherapy	24
28	Yu-Fei Gao et al. (34)	83	pelvic mass	uterine	–	R‐CHOP + local radiotherapy	–
29	Alexandra Martin et al. (35)	69	pelvic mass	uterine	Ann Arbor stage IE	R‐CHOP *4	–
30	Manoj Kumar Patro et al. (36)	62	Postmenopausal vaginal bleeding	uterine	–	CHOP *6+radiation	18
31	James Mega et al. (37)	61	pelvic mass	uterine	–	–	Died of disease progression two months after surgery
32	Suhua Shi et al. (38)	78	Postmenopausal vaginal bleeding	uterine	Ann Arbor stage II	–	loss to follow-up
33		53	Postmenopausal vaginal bleeding	uterine	Ann Arbor stage II	R-CDOP	33
34		70	Postmenopausal vaginal bleeding	uterine	Ann Arbor stage IV	total hysterectomy, bilateral adnexectomy, pelvic and para-aortic lymph node dissection, omentectomy+R-CDOP	32
35		70	pelvic mass, postmenopausal vaginal bleeding	Bilateral ovaries	Ann Arbor stage II	hysterectomy, bilateral adnexectomy and omentectomy+R-CHOP	102
36		58	postmenopausal vaginal bleeding	Cervix	Ann Arbor stage II	R-CHOP	98
37		33	pelvic mass lower abdominal pain	Bilateral ovaries	Ann Arbor stage IV	total hysterectomy, bilateral adnexectomy, pelvic and para-aortic lymph node dissection, omentectomy+R-CHOP	died of acute tumor lysis syndrome 1month after surgery
38		67	pelvic mass, abdominal distension	Bilateral ovaries	Ann Arbor stage II	total hysterectomy, bilateral adnexectomy, pelvic and para-aortic lymph node dissection, omentectomy+R-CHOP	79 months after surgery, recurrence occurred 48 months after surgery
39		73	pelvic mass	Left ovary	Ann Arbor stage IV	Left adnexectomy and lymphatic biopsy	Died a month after surgery
40		31	pelvic mass	Bilateral ovaries	Ann Arbor stage IV	hysterectomy, bilateral adnexectomy and omentectomy+R-CHOP	38
41		30	pelvic mass, lower abdominal pain, abdominal distension	Bilateral ovaries	Ann Arbor stage IV	total hysterectomy and bilateral adnexectomy+R-CHOP	31
42		58	pelvic mass, lower abdominal pain	Right ovary	Ann Arbor stage IV	total hysterectomy and bilateral adnexectomy+R-CHOP	22
43		72	pelvic mass, lower abdominal pain, abdominal distension	Bilateral ovaries	Ann Arbor stage IV	bilateral adnexectomy and lymphatic biopsy	died of COVID-19 3 month after surgery
44		77	postmenopausal vaginal bleeding, vaginal discharge	Cervix	Ann Arbor stage II	Radical total hysterectomy, bilateral adnexectomy and pelvic lymph node dissection+R-CHOP	9

### 3.2 Primary lymphoma of the uterine cervix

Uterine cervical lymphoma accounts for 0.008% of cervical malignancies (26). Its main clinical manifestations are vaginal bleeding and increased discharge. Uterine cervical exfoliative cytology is usually used as the primary screening test for cervical malignancy. However, it is difficult to diagnose cervical lymphoma, probably because lymphoma originates from the mesenchyme, which is covered by a layer of epithelial cells, and its anisotropic cells are not easy to obtain at an early stage (39, 40). Therefore, biopsy with immunohistochemistry is usually used to confirm the diagnosis of cervical lymphoma.

But no consensus has been reached regarding the best treatment of this disease due to its rarity. According to several studies, chemotherapy ± radiotherapy is the main treatment for primary diffuse large B cell lymphoma (DLBCL) of the cervix. This treatment can achieve complete remission with a 5-year survival rate of 80% (24, 29). For young female patients with fertility requirements, the treatment is usually chemotherapy + immunotherapy to reduce the damage to fertility caused by radiotherapy or surgery (41). Hysterectomy and radical surgery are not preferred and there is no evidence that surgical patients have a better prognosis (1, 6, 41). Treatment and prognosis of selected primary lymphoma of the uterine cervix are shown in **Table 3** (11–38).

### 3.3 Primary uterine lymphoma

Primary uterine lymphoma usually occurs in postmenopausal women. Its main clinical manifestations are postmenopausal vaginal bleeding, increased menstrual flow, and abnormal uterine bleeding. The uterus of patients with this type of disease usually enlarges progressively over a short period of time (42). Uterine lymphoma is diagnosed in the same way as cervical lymphoma - diagnostic curettage and endometrial biopsy. In the case of deeper tumours, the test result may be negative. Therefore, the diagnosis needs to be confirmed by a combination of clinical symptoms, imaging, laboratory, and hysteroscopy to exclude other disease diagnoses (43).

There is still no standard protocol for treating primary uterine lymphoma. Some studies have shown that surgery combined with chemotherapy has a better prognosis, while postoperative combined radiotherapy has a worse prognosis (44). Patients with primary diffuse large B cell lymphoma (DLBCL) can be treated with total hysterectomy with preservation of the ovaries and postoperative R-CHOP chemotherapy. In patients with fertility needs, if the diagnosis is clear before surgery, chemotherapy can be used to achieve clinical remission and avoid postoperative complications (6, 43). The prognosis of primary uterine lymphoma is poor, with a median survival of only 19.6 months and a 5-year survival rate of 25%, according to the literature (45). Treatment and prognosis of selected primary uterine lymphoma are shown in **Table 3** (11–38).

### 3.4 Primary vulvar lymphoma and vaginal lymphoma

Primary vulvar lymphoma and vaginal lymphoma are rare. Clinical manifestations usually include vaginal bleeding, prolapse of intravaginal masses, and formation of vulvar masses, which may be accompanied by local redness, swelling, pain, and ulceration. The standard treatment for vulvar and vaginal lymphoma is chemotherapy. The R-CHOP regimen is used in patients with diffuse large B cell lymphoma (DLBCL), and challenges remain in the treatment of relapsed lymphoma (46, 47).

## 4 Summary and outlook

Female genital lymphomas are relatively rare in clinical practice, and the clinical manifestations are not typical. In this paper, we present four cases who were admitted to the hospital with symptoms of increased adnexal mass, postmenopausal vaginal bleeding, and vulvar mass. The pathology suggested primary malignant genital tract lymphoma in the course of diagnosis and treatment, and the patients were treated with R-CHOP chemotherapy in our hospital after surgery. Their lymphomas are all in stable stage at present. Primary large B cell lymphoma in female genital tract was treated following individualized treatment plans through multidisciplinary consultation, taking into account the primary site, patient’s age, fertility requirements, physical condition, and other conditions. Chemotherapy with the R-CHOP regimen was the primary treatment, supplemented by surgical treatment. The surgical treatment aimed at reducing the tumour load, and radiotherapy was not recommended as the standard and preferred treatment option. The limitations of this study lie in the relative rarity of female genital lymphomas, which makes it difficult to conduct a prospective study. Besides, this study has a small sample size and acts as a retrospective study only. We reviewed these four cases systematically to improve our clinical understanding of this type of disease. This will help us reduce omission and misdiagnosis, facilitate early diagnosis and treatment, improve patients’ quality of life, and increase their survival rate during future diagnosis and treatment of this type of disease. If possible, joint multi-center large-sample data studies can be conducted in future to explore and clarify key clinical diagnostic and treatment points of female genital lymphomas, so as to enhance clinicians’ comprehensive understanding of this disease and its diagnosis and treatment effectiveness.
